# Updated risk prediction model for pancreaticoduodenectomy using data from the National Clinical Database in Japan

**DOI:** 10.1002/ags3.12883

**Published:** 2024-11-11

**Authors:** Masamichi Mizuma, Hideki Endo, Hiroyuki Yamamoto, Mitsuhiro Shimura, Masahiro Iseki, Michiaki Unno, Taro Oshikiri, Yoshihiro Kakeji, Ken Shirabe

**Affiliations:** ^1^ Department of Surgery Tohoku University Graduate School of Medicine Sendai Japan; ^2^ The Japanese Society of Gastroenterological Surgery Tokyo Japan; ^3^ Department of Healthcare Quality Assessment Graduate School of Medicine The University of Tokyo Tokyo Japan; ^4^ Database Committee The Japanese Society of Gastroenterological Surgery Tokyo Japan

**Keywords:** mortality, pancreaticoduodenectomy, postoperative complication, prediction, risk model

## Abstract

**Aim:**

Risk prediction models for mortality, severe postoperative complications, and postoperative pancreatic fistula in patients undergoing pancreaticoduodenectomy were established using data from the National Clinical Database more than a decade ago, and the surgical outcomes of pancreaticoduodenectomy have improved over the years. The aim of this study is to update the risk prediction model for pancreaticoduodenectomy using National Clinical Database data.

**Methods:**

Between 2019 and 2021, the data of 35 365 patients who underwent pancreaticoduodenectomy and who were registered in the National Clinical Database were retrospectively analyzed. According to the registration year, the patients were divided into two cohorts: the development cohort (2019–2020; *n* = 23 654) and the validation cohort (2021; *n* = 11 711). Logistic regression analyses were performed to create risk models for surgical mortality, severe postoperative complications, and grade C postoperative pancreatic fistula.

**Results:**

Overall, the rates of surgical mortality, severe postoperative complications, and grade C postoperative pancreatic fistula were 1.8%, 2.2%, and 1.3%, respectively. Logistic regression analyses revealed 28, 28, and 14 risk factors for surgical mortality, severe postoperative complications, and grade C postoperative pancreatic fistula, respectively. The area under the receiver operating characteristic curve of the risk model in the development cohort was 0.759 for surgical mortality, 0.712 for severe complications, and 0.699 for postoperative pancreatic fistula, comparable to the validation cohort. The calibration plots were favorable in both cohorts.

**Conclusion:**

The updated risk model for pancreaticoduodenectomy will be useful to predict surgical mortality, severe postoperative complications, and grade C postoperative pancreatic fistula.

## Graphical abstract

The calibration plots of the updated risk models for the endpoints were analyzed in each cohort. The predicted probability of each endpoint matched the observed probability in the development and validation cohorts.

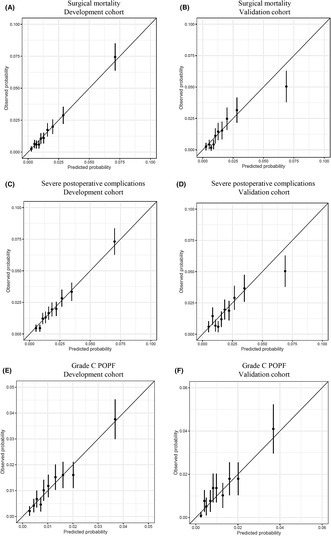

## 1 INTRODUCTION

Pancreaticoduodenectomy (PD), which is most frequently performed in patients with periampullary cancer, is a highly invasive surgical procedure. The surgical mortality rate of PD, which is reportedly 1.5%–2.9%, is higher than that of other gastrointestinal surgical procedures.^1^, ^2^, ^3^, ^4^ Preoperative risk factors for surgical mortality in patients undergoing PD, such as hypertension, history of cardiac surgery, age, bleeding, low serum albumin, disseminated cancer, steroid use, systemic inflammatory response syndrome, respiratory distress, activities of daily living (ADL) within 30 d before surgery, body weight loss, and high serum creatinine, have been reported previously.^1^, ^2^ Risk assessment considering these risk factors before PD is essential in the clinical setting. In Japan, the risk calculator for the main abdominal procedures in the National Clinical Database (NCD), which is based on risk models that have been established using nationwide data from the NCD, enables the prediction of surgical mortality and complications.^1^, ^5^ The mortality risk model for PD, which was reported in 2014, was created using the data of patients who underwent PD between January 2011 and December 2011.^1^ Furthermore, the PD risk models for severe postoperative complications and grade C postoperative pancreatic fistula (POPF), which were constructed using former POPF criteria, were established using NCD data from 2011 to 2012.^6^

The NCD, which began in 2011, is a patient registration system in Japan. More than 95% of all surgical cases in Japan are registered in the NCD every year. Recently, more than 10 000 PD cases per year have been registered in the NCD.^7^ To date, NCD data have been used in a wide variety of studies, such as studies constructing risk prediction models for major surgical procedures, studies identifying quality indicators, and studies evaluating the impact of coronavirus disease 2019 on surgical procedures.^1^, ^8^, ^9^, ^10^, ^11^, ^12^, ^13^, ^14^

Surgical mortality and morbidity from PD have declined over the years. In the report of the NCD risk model, the surgical mortality rate from PD was 2.8% in 2011.^1^ The mortality rate from PD has gradually decreased, and was reportedly 1.8% in 2019 according to the NCD data, while the number of PD procedures has gradually increased.^7^ The surgical technique, perioperative management, and treatment for preoperative comorbidity are continuously improving. Recently, neoadjuvant therapy has been performed standardly in patients with pancreatic cancer.^15^ Therefore, it is optimal to create risk models to predict surgical outcomes using new data, namely “real data.” Predictive risk models constructed from NCD data have been refined in esophagectomy, low anterior resection, and acute diffuse peritonitis in recent times.^16^, ^17^, ^18^ This study aims to update the risk prediction model for PD using the latest NCD data.

## 2 METHODS

### 2.1 Study design

**FIGURE 1:**
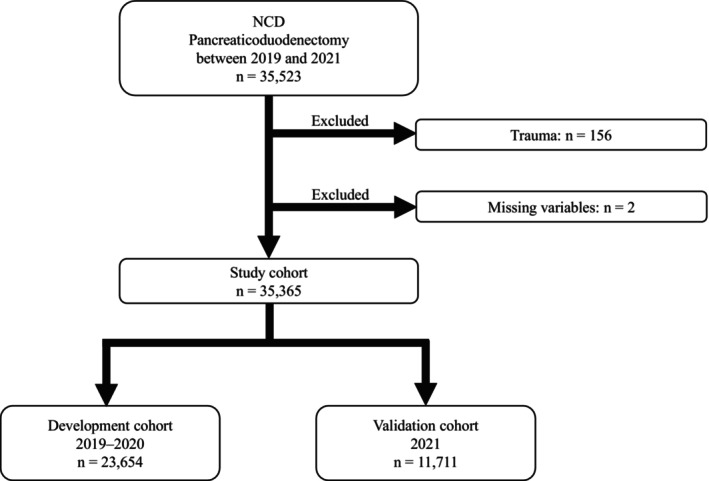
Flowchart of patient selection. Patients who underwent hepatopancreaticoduodenectomy were included in the study cohort.

The NCD data were collected using a web‐based system on the NCD server and verified through site visit audits to randomly selected institutions. The NCD data registered between 2019 and 2021 were retrospectively reviewed. Of the 35 523 patients who underwent PD, 156 were excluded for trauma and two were excluded due to missing variables. Finally, 35 365 patients were included. The patients were divided into two cohorts: the development cohort (2019–2020; *n* = 23 654) and the validation cohort (2021; *n* = 11 711) (Figure 1). Patients who underwent hepatopancreaticoduodenectomy (HPD) were included in the study cohort. The definitions of all variables are accessible from the participating institutions on the NCD website (http://www.ncd.or.jp/).

The protocol for this research project was approved by the Japanese Society of Gastroenterological Surgery and the Ethics Committee of Kobe University (approval no. 20190128) and it conforms to the provisions of the Declaration of Helsinki. The requirement for written informed consent was waived because of the retrospective study design and the use of anonymized clinical data.

### 2.2 Endpoints

The primary endpoint was surgical mortality after PD, which was defined as 30‐d mortality, including death after discharge or in‐hospital mortality. The secondary endpoints were severe postoperative complications and grade C POPF according to the criteria of the International Study Group on Pancreatic Surgery (ISGPS).^19^ Severe postoperative complications were defined as Clavien–Dindo classification grade 4 or higher.^20^

### 2.3 Risk factors

A total of 74 preoperative variables evaluated as candidate risk factors were as follows: age, sex, preoperative therapy, body mass index (BMI), lifestyle habits (smoking and alcohol consumption), various preoperative comorbidities, history of surgery for cardiovascular disease, preoperative anticoagulation therapy, preoperative laboratory data, and the American Society of Anesthesiologists physical status (ASA‐PS), among others (Table 1). In addition, the types and tumor–node–metastasis (TNM) characteristics of malignant diseases were evaluated as candidate risk factors (Table 2). Gastric cancer, cancer of the small intestine, well‐differentiated pancreatic neuroendocrine neoplasm G1 and G2, hepatocellular carcinoma, intrahepatic cholangiocarcinoma, perihilar cholangiocarcinoma, distal cholangiocarcinoma, gallbladder carcinoma, cancer of the ampulla of Vater, and pancreatic cancer were analyzed. TNM factors were intraoperatively determined according to the TNM Classification 8th edition of the Union for International Cancer Control.^21^ T, N, and M factors in each malignancy were evaluated in the risk analyses. These variables were chosen and included in the regression model based on subject matter knowledge and previous research. All of the variables were entered as a dichotomous variable. For example, if the variable is T4 for pancreatic cancer, the reference level is other than T4 for pancreatic cancer, including benign diseases.

**TABLE 1:** Preoperative baseline characteristics for the study population.

Preoperative variables	Development cohort *n* = 23 654	Validation cohort *n* = 11 711
Age (years)		
≥60	20 571 (87.0%)	10 196 (87.1%)
≥70	14 612 (61.8%)	7538 (64.4%)
≥80	3741 (15.8%)	1951 (16.7%)
Sex, male	14 340 (60.6%)	7053 (60.2%)
Emergency surgery	133 (0.6%)	78 (0.7%)
Preoperative chemotherapy within 90 d	3993 (16.9%)	2918 (24.9%)
Preoperative radiotherapy within 90 d	506 (2.1%)	263 (2.2%)
Preoperative immunotherapy within 90 d	28 (0.1%)	17 (0.1%)
BMI (kg/m^2^)		
<18.5	2950 (12.5%)	1474 (12.6%)
≥25	4339 (18.3%)	2130 (18.2%)
≥30	533 (2.3%)	270 (2.3%)
Diabetes mellitus	7576 (32.0%)	3739 (31.9%)
Smoking within 1 year	4276 (18.1%)	1959 (16.7%)
Brinkman index ≥400	8321 (35.2%)	4031 (34.4%)
Habitual alcohol consumption	6815 (28.8%)	3373 (28.8%)
Dyspnea	211 (0.9%)	73 (0.6%)
ADL: any assistance		
Within 30 d	536 (2.3%)	299 (2.6%)
Just before surgery	457 (1.9%)	257 (2.2%)
On a ventilator within 48 h	16 (0.1%)	12 (0.1%)
Chronic obstructive pulmonary disease	813 (3.4%)	373 (3.2%)
Pneumonia	54 (0.2%)	32 (0.3%)
Encephalopathy within 30 d	21 (0.1%)	3 (0.0%)
Ascites within 30 d	268 (1.1%)	127 (1.1%)
Esophageal varices within 6 mo	45 (0.2%)	24 (0.2%)
Hypertension within 30 d	10 434 (44.1%)	5418 (46.3%)
Congestive heart failure within 30 d	70 (0.3%)	39 (0.3%)
Myocardial infarction within 6 mo	68 (0.3%)	30 (0.3%)
Angina pectoris within 30 d	234 (1.0%)	120 (1.0%)
Past history		
Percutaneous coronary intervention	718 (3.0%)	342 (2.9%)
Cardiac surgery	275 (1.2%)	120 (1.0%)
Surgery for peripheral vascular disease	93 (0.4%)	46 (0.4%)
Symptomatic peripheral vascular disease	54 (0.2%)	39 (0.3%)
Acute renal failure within 24 h	11 (0.0%)	6 (0.1%)
Preoperative hemodialysis within 14 d	135 (0.6%)	64 (0.5%)
History of cerebrovascular disease	1043 (4.4%)	535 (4.6%)
Multiple tumor metastases	68 (0.3%)	33 (0.3%)
Open wound just before surgery	14 (0.1%)	7 (0.1%)
Long‐term steroid use	371 (1.6%)	162 (1.4%)
Body weight loss (≥10% in the past 6 mo)	1091 (4.6%)	565 (4.8%)
Anticoagulation therapy	2716 (11.5%)	1451 (12.4%)
Bleeding risk factors	726 (3.1%)	352 (3.0%)
Blood transfusion within 72 h	206 (0.9%)	121 (1.0%)
Preoperative chemotherapy within 30 d	1773 (7.5%)	1424 (12.2%)
Sepsis	85 (0.4%)	16 (0.1%)
Preoperative laboratory data		
WBC count ≥11 000/μL	500 (2.1%)	253 (2.2%)
Hemoglobin (g/dL)		
<8	184 (0.8%)	84 (0.7%)
Male <11, female <10	3355 (14.2%)	1687 (14.4%)
Male <13.5, female <11.5	11 846 (50.1%)	6096 (52.1%)
Hematocrit, male ≥48%, female ≥42%	407 (1.7%)	228 (1.9%)
Platelet count (/μL)		
<120 000	641 (2.7%)	314 (2.7%)
<80 000	97 (0.4%)	40 (0.3%)
Serum albumin (g/dL)		
<2.0	79 (0.3%)	31 (0.3%)
<2.8	1101 (4.7%)	522 (4.5%)
<3.5	5951 (25.2%)	2842 (24.3%)
Serum total bilirubin (mg/dL)		
≥2.0	2975 (12.6%)	1232 (10.5%)
>3.0	1646 (7.0%)	724 (6.2%)
Serum AST >40 U/L	5161 (21.8%)	2342 (20.0%)
Serum ALT >40 U/L	6765 (28.6%)	3032 (25.9%)
Serum BUN >20 mg/dL	2739 (11.6%)	1408 (12.0%)
eGFR (mL/min/1.73 m^2^)		
<45	1533 (6.5%)	748 (6.4%)
<30	418 (1.8%)	198 (1.7%)
<15	193 (0.8%)	93 (0.8%)
Serum sodium >146 mEq/L	92 (0.4%)	27 (0.2%)
HbA1c (%)		
≥6.5	5433 (23.0%)	3094 (26.4%)
≥7.0	3705 (15.7%)	2133 (18.2%)
≥8.0	1800 (7.6%)	962 (8.2%)
Serum CRP ≥1.0 mg/dL	3674 (15.5%)	1738 (14.8%)
APTT ≥40 s	673 (2.8%)	302 (2.6%)
PT‐INR		
>1.1	1912 (8.1%)	921 (7.9%)
≥1.7	100 (0.4%)	49 (0.4%)
>2.3	35 (0.1%)	12 (0.1%)
ASA‐PS grade		
≥3	3570 (15.1%)	1853 (15.8%)
≥4	63 (0.3%)	33 (0.3%)
5	18 (0.1%)	12 (0.1%)

Abbreviations: ADL, activities of daily living; ALT, alanine aminotransferase; APTT, activated partial thromboplastin time; ASA‐PS, American Society of Anesthesiologists physical status; AST, aspartate aminotransferase; BMI, body mass index; BUN, blood urea nitrogen; CRP, C‐reactive protein; eGFR, estimated glomerular filtration rate; HbA1c, hemoglobin A1c; PT‐INR, prothrombin time‐international normalized ratio; WBC, white blood cell.

**TABLE 2:** Primary tumors, intraoperative TNM stage, and postoperative outcomes.

Characteristics	Development cohort *n* = 23 654	Validation cohort *n* = 11 711
Malignancy		
Gastric cancer	295 (1.2%)	142 (1.2%)
T4b	65 (0.3%)	35 (0.3%)
N1–3	126 (0.5%)	68 (0.6%)
M1	22 (0.1%)	13 (0.1%)
Cancer of the small intestine	801 (3.4%)	388 (3.3%)
T4	259 (1.1%)	125 (1.1%)
N1–2	373 (1.6%)	173 (1.5%)
M1	39 (0.2%)	17 (0.1%)
Well‐differentiated PanNEN (G1 and G2)	637 (2.7%)	302 (2.6%)
T4	10 (0.0%)	5 (0.0%)
N1	128 (0.5%)	58 (0.5%)
M1a–c	19 (0.1%)	12 (0.1%)
Hepatocellular carcinoma	22 (0.1%)	4 (0.0%)
T4	0 (0.0)	0 (0.0)
N1	1 (0.0)	0 (0.0)
M1	0 (0.0)	0 (0.0)
Intrahepatic cholangiocarcinoma	40 (0.2%)	13 (0.1%)
T4	1 (0.0%)	0 (0.0)
N1	14 (0.1%)	3 (0.0%)
M1	0 (0.0%)	0 (0.0)
Perihilar cholangiocarcinoma	339 (1.4%)	165 (1.4%)
T4	9 (0.0%)	6 (0.1%)
N1–2	122 (0.5%)	65 (0.6%)
M1	4 (0.0%)	3 (0.0%)
Distal cholangiocarcinoma	4420 (18.7%)	2210 (18.9%)
T4	33 (0.1%)	31 (0.3%)
N1–2	1644 (7.0%)	807 (6.9%)
M1	59 (0.2%)	28 (0.2%)
Gallbladder carcinoma	170 (0.7%)	73 (0.6%)
T4	20 (0.1%)	9 (0.1%)
N1–2	88 (0.4%)	43 (0.4%)
M1	11 (0.0%)	5 (0.0%)
Cancer of the ampulla of Vater	2479 (10.5%)	1196 (10.2%)
T4	43 (0.2%)	11 (0.1%)
N1–2	809 (3.4%)	379 (3.2%)
M1	40 (0.2%)	18 (0.2%)
Pancreatic cancer	12 081 (51.1%)	6162 (52.6%)
T4	239 (1.0%)	105 (0.9%)
N1–2	5434 (23.0%)	2612 (22.3%)
M1	298 (1.3%)	138 (1.2%)
Postoperative outcomes		
Surgical mortality	432 (1.8%)	188 (1.6%)
Severe postoperative complications	533 (2.3%)	234 (2.0%)
Grade C POPF	295 (1.2%)	159 (1.4%)

Abbreviations: PanNEN, pancreatic neuroendocrine neoplasm; POPF, postoperative pancreatic fistula.

### 2.4 Statistical analysis

Data analysis was performed using R, v. 4.2.2 (2022; R Foundation for Statistical Computing, Vienna, Austria). Risk models for each outcome were created by logistic regression analysis using the backward stepwise variable selection method determined by the Akaike information criterion. As previously described, according to the logistic regression equation, the risk prediction formula was as follows: predicted outcomes = e (β0 + ∑βiXi)/1 + e (β0 + ∑βiXi), where βi is the coefficient of the variable Xi in the logistic regression equation.^22^ Model performance was assessed by the area under the receiver operating characteristic curve (AUROC). For model calibration, the predicted probability was compared with the observed probability for each outcome with a decile calibration plot. *p* < 0.05 was considered statistically significant.

## 3 RESULTS

### 3.1 Clinical characteristics and surgical outcomes of the study population

The preoperative baseline characteristics of the study population are shown in Table 1. Most of the variables were similar between the development and validation cohorts. Approximately one‐sixth of the patients were aged ≥80 y. Compared with the development cohort, the proportion of patients receiving preoperative chemotherapy within 30 or 90 d was higher in the validation cohort.

Pancreatic cancer was the most common type of malignancy, followed by distal cholangiocarcinoma and cancer of the ampulla of Vater (Table 2). Patients with T4 and N1‐2 pancreatic cancer accounted for 1% and 23% of the study cohort, respectively. The type of malignancy and candidate risk factors by TNM stage were comparable between the development and validation cohorts.

The surgical mortality rate was 1.8% and 1.6% in the development and validation cohorts, respectively (Table 2). Severe postoperative complications occurred in 2.3% of the patients in the development cohort and 2.0% in the validation cohort. The complication rate of grade C POPF was 1.2% in the development cohort and 1.4% in the validation cohort.

### 3.2 Logistic regression analysis to establish the risk model

Table 3 shows the results of the logistic regression analysis for each endpoint. For surgical mortality, age ≥60, ≥70, and ≥80 y; BMI ≥25 kg/m^2^; male sex; dyspnea; ADL requiring assistance within 30 d; history of cardiac surgery; long‐term steroid use; platelet count <120 000/μL; serum albumin <2.8 and <3.5 (g/dL); serum AST >40 U/L; estimated glomerular filtration rate (eGFR) <30 mL/min/1.73 m^2^; serum C‐reactive protein (CRP) ≥1.0 mg/dL; cancer of the small intestine; distal extrahepatic bile duct cancer; gallbladder cancer (T4); and pancreatic cancer (T4) were significant independent risk factors. Among them, gallbladder cancer (T4) had the highest odds ratio (OR; 7.581) (*p* = 0.004), which was calculated using the reference level of other than gallbladder cancer (T4), including benign and other malignant diseases. M1 in each malignancy was not a significant predictor for surgical mortality.

**TABLE 3:** Logistic regression models for surgical mortality, severe postoperative complications, and grade C POPF.

Variables	OR	95% CI	*p* value
Surgical mortality			
Intercept	0.002	0.001–0.003	<0.001
Age (years)			
≥60	2.556	1.433–4.560	0.001
≥70	1.597	1.218–2.093	<0.001
≥80	1.402	1.110–1.770	0.005
Sex, male	1.495	1.205–1.856	<0.001
BMI (kg/m^2^)			
≥25	1.845	1.457–2.336	<0.001
≥30	1.564	0.911–2.685	0.105
Dyspnea	2.277	1.289–4.023	0.005
ADL, any assistance within 30 d	2.104	1.445–3.065	<0.001
History of cardiac surgery	2.734	1.665–4.489	<0.001
Long‐term steroid use	2.772	1.723–4.460	<0.001
Body weight loss (≥10% in the past 6 mo)	1.354	0.933–1.963	0.110
Blood transfusion within 72 h	1.733	0.963–3.122	0.067
Sepsis	2.021	0.878–4.652	0.098
Platelet count <120 000/μL	2.012	1.355–2.987	<0.001
Serum albumin (g/dL)			
<2.8	1.747	1.272–2.400	<0.001
<3.5	1.403	1.108–1.777	0.005
Serum AST >40 U/L	1.374	1.102–1.713	0.005
eGFR <30 mL/min/1.73 m^2^	2.378	1.547–3.656	<0.001
Serum CRP ≥1.0 mg/dL	1.468	1.159–1.858	0.001
PT‐INR >1.1	1.277	0.968–1.684	0.084
ASA‐PS grade			
≥3	1.262	0.998–1.597	0.052
≥4	2.380	0.979–5.787	0.056
Malignancy			
Gastric cancer	1.810	0.967–3.386	0.064
Cancer of the small intestine	2.138	1.398–3.269	<0.001
Intrahepatic cholangiocarcinoma	3.270	0.955–11.200	0.059
Distal extrahepatic bile duct	1.339	1.063–1.686	0.013
TNM classification			
Gallbladder cancer T4	7.581	1.895–30.326	0.004
Pancreatic cancer T4	2.827	1.438–5.557	0.003
Severe postoperative complications			
Intercept	0.005	0.003–0.007	<0.001
Age (years)			
≥60	2.044	1.331–3.140	0.001
≥70	1.320	1.052–1.658	0.017
≥80	1.231	0.985–1.538	0.067
Sex, male	1.764	1.446–2.152	<0.001
BMI (kg/m^2^)			
≥25	1.844	1.497–2.272	<0.001
≥30	1.641	1.039–2.592	0.034
Dyspnea	2.162	1.270–3.681	0.005
ADL, any assistance within 30 d	1.992	1.377–2.882	<0.001
Hypertension within 30 d	1.148	0.959–1.375	0.133
History of cardiac surgery	1.718	1.017–2.903	0.043
Long‐term steroid use	2.414	1.531–3.804	<0.001
Body weight loss (≥10% in the past 6 mo)	1.390	0.982–1.967	0.063
Platelet count <120 000/μL	1.521	1.022–2.264	0.039
Serum albumin (g/dL)			
<2.0	1.925	0.852–4.348	0.115
<2.8	1.504	1.067–2.119	0.020
<3.5	1.219	0.989–1.502	0.063
eGFR (mL/min/1.73 m^2^)			
<45	1.328	0.954–1.849	0.093
<30	1.921	1.178–3.130	0.009
APTT ≥40 s	1.670	1.156–2.413	0.006
ASA‐PS grade			
≥3	1.286	1.036–1.595	0.023
≥4	2.236	0.937–5.336	0.070
Malignancy			
Well‐differentiated PanNEN (G1 and G2)	0.592	0.289–1.213	0.152
Cancer of the ampulla of Vater	0.552	0.368–0.829	0.004
Pancreatic cancer	0.708	0.586–0.855	<0.001
TNM classification			
Well‐differentiated PanNEN (G1 and G2) T4	8.461	0.906–78.984	0.061
Gallbladder cancer N1–2	2.839	1.266–6.370	0.011
Cancer of the ampulla of Vater N1–2	1.575	0.871–2.848	0.133
Pancreatic cancer T4	1.792	0.861–3.729	0.119
Grade C POPF			
Intercept	0.004	0.003–0.007	<0.001
Age (years)			
≥60	1.932	1.227–3.042	0.004
Sex, male	1.548	1.170–2.048	0.002
BMI (kg/m^2^)			
<18.5	0.718	0.450–1.146	0.165
≥25	2.118	1.648–2.723	<0.001
Habitual alcohol consumption	1.211	0.940–1.562	0.139
Long‐term steroid use	3.046	1.714–5.414	<0.001
Anticoagulation therapy	1.419	1.045–1.927	0.025
Preoperative chemotherapy within 30 d	0.480	0.223–1.033	0.061
Hematocrit (≥48% in male, ≥42% in female patients)	1.801	0.875–3.707	0.110
Serum albumin (g/dL)			
<2.0	2.971	0.967–9.126	0.057
<2.8	1.571	0.977–2.525	0.062
eGFR (mL/min/1.73 m^2^)			
<45	1.626	1.135–2.329	0.008
Malignancy			
Distal cholangiocarcinoma	1.301	0.982–1.723	0.066
Pancreatic cancer	0.545	0.409–0.725	<0.001

Abbreviations: ADL, activities of daily living; APTT, activated partial thromboplastin time; ASA‐PS, American Society of Anesthesiologists physical status; AST, aspartate aminotransferase; BMI, body mass index; CRP, C‐reactive protein; eGFR, estimated glomerular filtration rate; PanNEN, pancreatic neuroendocrine neoplasm; POPF, postoperative pancreatic fistula; PT‐INR, prothrombin time‐international normalized ratio.

For severe postoperative complications, age ≥60 and ≥70 y; male sex; BMI ≥25 and ≥30 kg/m^2^; dyspnea; ADL requiring assistance within 30 d; history of cardiac surgery; long‐term steroid use; platelet count <120 000/μL; serum albumin <2.8 g/dL; eGFR <30 mL/min/1.73 m^2^; activated partial thromboplastin time ≥40 s; ASA‐PS grade ≥3; cancer of the ampulla of Vater; pancreatic cancer; and gallbladder cancer N1–2 were significant independent risk factors. Among them, gallbladder cancer N1–2 had the highest OR (2.839) (*p* = 0.011), followed by long‐term steroid use (OR 2.414, *p* < 0.001).

The independent risk factors for grade C POPF were age ≥60 y, male sex, BMI ≥25 kg/m^2^, long‐term steroid use, anticoagulation therapy, eGFR <45 mL/min/1.73 m^2^, and nonpancreatic cancer. Pancreatic cancer had an OR of 0.545 (*p* < 0.001).

### 3.3 Model performance and calibration

To confirm the model performance, the ROC curves in the development and validation cohorts were evaluated for each outcome. The AUROC and 95% confidence interval (CI) for each outcome are shown in Table 4. For surgical mortality, the AUROC value was 0.759 and 0.754 in the development and validation cohorts, respectively. Severe postoperative complications had AUROC values of 0.712 and 0.689 in the development and validation cohorts, respectively. The AUROC values for grade C POPF were 0.699 and 0.692 in the development and validation cohorts, respectively.

**TABLE 4:** Model performance.

	AUROC	95% CI
Surgical mortality		
Development cohort	0.759	0.736–0.783
Validation cohort	0.754	0.720–0.786
Severe postoperative complications		
Development cohort	0.712	0.690–0.735
Validation cohort	0.689	0.654–0.724
Grade C POPF		
Development cohort	0.699	0.670–0.728
Validation cohort	0.692	0.652–0.733

Abbreviations: AUROC, area under the receiver operator characteristic curve; CI, confidence interval; POPF, postoperative pancreatic fistula.

**FIGURE 2:**
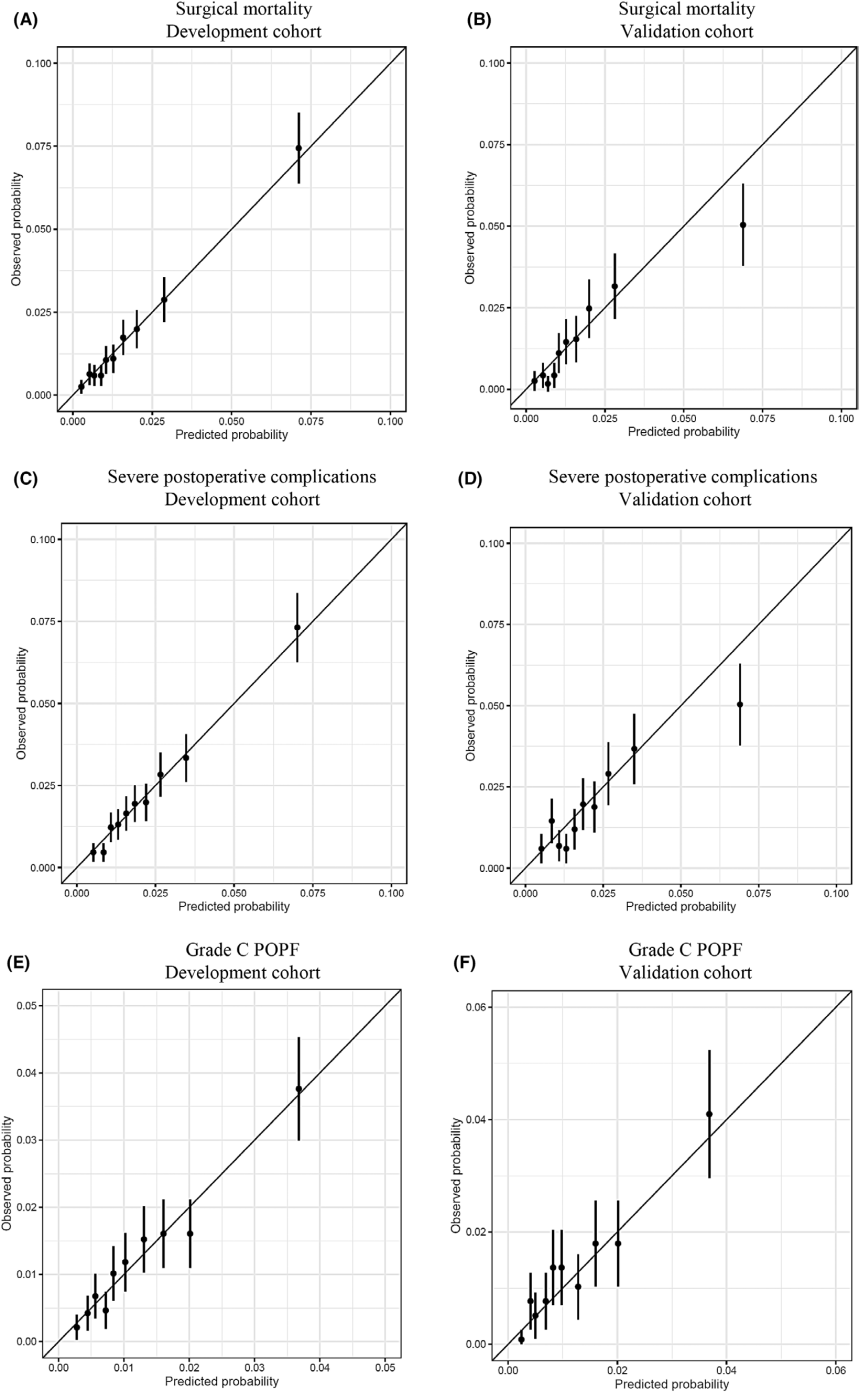
Calibration plot for each outcome in the development and validation cohorts. (A) Surgical mortality in the development cohort. (B) Surgical mortality in the validation cohort. (C) Severe postoperative complications in the development cohort. (D) Severe postoperative complications in the validation cohort. (E) Grade C POPF in the development cohort. (F) Grade C POPF in the validation cohort. POPF, postoperative pancreatic fistula.

The calibration plots of the established risk models for the endpoints were analyzed in each cohort (Figure 2). The predicted probability of each endpoint matched the observed probability in the development and validation cohorts. Therefore, the risk prediction model was considered acceptable for all endpoints.

## 4 DISCUSSION

This study updated the risk prediction model of PD for surgical mortality, severe surgical complications, and grade C POPF using NCD data between 2019 and 2021. TNM stage, which was not included in the previous risk prediction models, was added as a variable in this study. Although the previous model was created according to the former definition of POPF, the model of grade C POPF was updated using the current definition. The present model was confirmed as being acceptable through the validation analysis. This updated risk prediction model will be useful in the clinical setting and can be used as a risk calculator by NCD users.

The surgical outcomes of PD are continuously improving, and classifications related to surgery have been revised over time. Thus, risk prediction models should be refined regularly using real‐world data, such as data from the NCD. In previous risk prediction models, the rates of in‐hospital mortality, severe postoperative complications, and grade C POPF were 2.88%, 4.45%, and 4.83%, respectively.^1^, ^5^ The results of this study showed improved outcomes, with surgical mortality rates of 1.6%–1.8%, severe postoperative complication rates of 2.0%–2.3%, and grade C POPF rates of 1.2–1.4%. Although the decline in the rate of grade C POPF might be partly due to the change in the definition of POPF, the improvement in the surgical outcomes of PD is thought to be due to the optimization of surgical techniques and perioperative management. In particular, the development of radiological interventions and endoscopic treatments has hugely impacted the postoperative management of patients undergoing PD.^23^ Moreover, risk calculators have played a vital role in these outcome improvements.^16^ A risk calculator has been installed on the NCD website, allowing NCD users, almost all of whom are surgeons in Japan, to calculate a patient's predicted rate of mortality, severe complications, and grade C POPF using preoperative clinical data. There may be some patients for whom the results of the risk calculator have influenced the decision to perform surgery. In other words, some patients with a high risk of mortality predicted by the risk calculator may be deemed ineligible for surgery. Thus, the risk calculator may contribute to improving surgical outcomes.

The AUROC value was calculated to evaluate the model performance. In previous models, the AUROC values for in‐hospital mortality, severe postoperative complications, and grade C POPF were 0.725, 0.708, and 0.700, respectively. In the present model, the AUROC value for surgical mortality was 0.759 in the development cohort and 0.754 in the validation cohort, higher than the value of the previous model. The AUROC values for severe postoperative complications and grade C POPF in the present model were equivalent to those of the previous model. In addition, the calibration plots for surgical mortality, severe postoperative complications, and grade C POPF demonstrated that the predicted probability matched the observed probability in both the development and validation cohorts. Therefore, the present model exhibited comparable performance to the previous model and would be acceptable in the clinical setting.

Pancreatic cancer is in the era of preoperative therapy.^24^, ^25^ In the present study, the proportion of patients treated with preoperative chemotherapy within 90 d was 16.9% in the development cohort and 24.9% in the validation cohort, increasing over time. Although preoperative therapy was not considered in the previous models, it was evaluated as a candidate risk factor in this study. Preoperative chemotherapy was not a risk factor for surgical mortality and severe postoperative complications. However, preoperative chemotherapy within 30 d was a favorable factor for grade C POPF, with an OR of 0.480 (95% CI 0.223–1.033, *p* = 0.061). Furthermore, although pancreatic cancer was not considered as a risk factor in the previous model, pancreatic cancer was one of the favorable risk factors in the present model for grade C POPF (OR 0.545, 95% CI 0.409–0.725, *p* < 0.001). Most patients with preoperative chemotherapy within 30 d presumably had pancreatic cancer. The reason pancreatic cancer is a favorable risk factor for grade C POPF is that, compared with other malignancies, patients with pancreatic cancer more frequently have a pancreas with a hard texture.^26^, ^27^ Moreover, pancreatic cancer was a favorable risk factor for severe postoperative complications. This might be related to the results showing that pancreatic cancer was a favorable risk factor for grade C POPF.

TNM stage, which affects the extent of resection and surgical invasiveness in hepatobiliary pancreatic malignancies, was not considered in the previous risk prediction model. For this reason, the present risk prediction model was constructed considering the TNM stage. T4 gallbladder cancer (OR 7.581, 95% CI 1.895–30.326, *p* = 0.004) and T4 pancreatic cancer were significant risk factors for surgical mortality. Additionally, N1–2 gallbladder cancer was a significant risk factor for severe postoperative complications. This is because patients with these conditions often require highly invasive surgeries, such as combined vascular resection and reconstruction for curative resection. Notably, patients with highly advanced gallbladder cancer might undergo HPD, having high mortality and morbidity rates of 11.4% and 72.5%, respectively, according to NCD data.^28^

In this study, intraoperative TNM findings were evaluated in the logistic regression analyses. Preoperative TNM findings may not match intraoperative TNM findings. Therefore, in the case of preoperative risk prediction using preoperative TNM findings, this characteristic of the updated model should be understood.

The main limitation of this study is that the NCD data are from patients who underwent surgery at Japanese hospitals only; therefore, data from hospitals in other countries were not included. This may limit the usefulness of this risk prediction model for PD in other countries.

In conclusion, we updated the risk prediction model for PD using data from the NCD. The performance of the updated model is comparable to that of the previous model, and it will be useful for the prediction of surgical mortality, severe postoperative complications, and grade C POPF in the clinical setting.

## AUTHOR CONTRIBUTIONS

**Masamichi Mizuma:** Conceptualization; formal analysis; writing – original draft. **Hideki Endo:** Conceptualization; data curation; formal analysis; methodology; writing – review and editing. **Hiroyuki Yamamoto:** Conceptualization; data curation; formal analysis; methodology. **Mitsuhiro Shimura:** Writing – review and editing. **Masahiro Iseki:** Writing – review and editing. **Michiaki Unno:** Supervision. **Taro Oshikiri:** Project administration; supervision. **Yoshihiro Kakeji:** Project administration; supervision. **Ken Shirabe:** Supervision.

## FUNDING INFORMATION

The funding for this study was provided by the Japanese Society of Gastroenterological Surgery (APJ–2019–02).

## CONFLICT OF INTEREST STATEMENT

Hideki Endo and Hiroyuki Yamamoto are affiliated with the Department of Healthcare Quality Assessment at the University of Tokyo, which is a social collaboration department supported by grants from the National Clinical Database, Intuitive Surgical Sarl, Johnson and Johnson K.K., and Nipro Co. Yoshihiro Kakeji and Ken Shirabe are editorial members of the *Annals of Gastroenterological Surgery*.

## ETHICS STATEMENT

Approval of the research protocol by an Institutional Reviewer Board: The protocol for this research project was approved by the Japanese Society of Gastroenterological Surgery and the Ethics Committee of Kobe University (approval no. 20190128).

Informed Consent: N/A.

Registry and the Registration No. of the study/trial: N/A.

Animal Studies: N/A.

## Data Availability

Research data are not shared. Data from the National Clinical Database (NCD) in Japan was used in this study.
